# International norms and the politics of sexuality education in Nigeria

**DOI:** 10.1186/s12992-018-0377-2

**Published:** 2018-07-03

**Authors:** Jeremy Shiffman, Michael Kunnuji, Yusra Ribhi Shawar, Rachel Sullivan Robinson

**Affiliations:** 1 Department of Public Administration and Policy, School of Public Affairs, American University, 4400 Massachusetts Ave., NW, Washington, DC 20016-8070 USA; 2 Department of Sociology, University of Lagos, Lagos, Nigeria; 3 School of International Service, American University, Washington, DC, USA

**Keywords:** Nigeria, Reproductive health, HIV/AIDS, Adolescents, International relations theory, Health education, Sexuality education, Policy process, Policy analysis, NGOs

## Abstract

**Background:**

Proponents have promoted sexuality education as a means of empowering adolescents, yet it has been thwarted in many low and middle-income countries. Nigeria represents an exception. Despite social opposition, the government in 1999 unexpectedly approved sexuality education policy. Since then, implementation has advanced, although efficacy has differed across states. We draw on theory concerning international norm diffusion to understand Nigerian policy development.

**Results:**

We find that a confluence of international and national norms and interests shaped policy outcomes, including concern over HIV/AIDS. A central dynamic was an alliance of domestic NGOs and international donors pressing the Nigerian government to act.

**Conclusions:**

We argue that theory on international norms can be applied to understand policy dynamics across a variety of health and population areas, finding value in approaches that integrate rather than juxtapose consideration of (1) international and national influences; (2) long and short-term perspectives on policy change; and (3) norms and interests.

## Background

Adolescents face considerable reproductive risks. Globally, seventy thousand die each year due to complications from pregnancy [1]. In 2015, 20% of new HIV infections to people 15 and over occurred among adolescent girls and young women aged 15 to 24, even as they comprised only 11% of that population [2]. Fourteen percent of new HIV infections to people 15 and over occurred among adolescent boys and young men aged 15 to 24 [2]. These adverse outcomes are a result of the precarious conditions adolescents face—reflections of “powerlessness, poverty and pressures—from partners, peers, families and communities” (p. vii) [1].

The reproductive health and rights of adolescents are a growing global priority. The Sustainable Development Goals include explicit mention of adolescents, and the United Nations has produced a global strategy for women’s, children’s and adolescents’ health [3]. Proponents have argued for school-based sexuality education as one means of empowering adolescents to gain control over their bodies and lives, and to improve reproductive health outcomes. While research on program design and effects is extensive [4–7], there are few studies of the politics of program adoption and implementation in low and middle-income settings (see [8] for an exception). Understanding political dynamics is crucial, since sexuality education incites opposition, making adoption and implementation complex.

This paper, part of a larger research project on managing the politics of sexuality education, examines these political dynamics in Nigeria. Nigeria is a revealing case for several reasons. It has one of the largest populations of at-risk adolescents in the world [9]. In addition, it stands as one of the few low-income countries outside Latin America that has managed to make sexuality education available to a sizeable proportion of in-school adolescents [10] and has done so despite social norms that mitigate against school-based delivery. Factors that led to prioritization may also be present in other countries, so understanding Nigeria may help us understand how to promote sexuality education elsewhere.

In studying this case, we draw on international relations and sociological scholarship pertaining to the power of international norms to shape national policy-making. Some scholars find extensive influence [11]. Others disagree, pointing to heavy influence of local and national processes [12] or finding that interests rather than norms drive policy-making [13]. These debates are directly germane to understanding policy dynamics in several health and population areas where both international and local influences potentially shape policy development.

In the sections that follow, we consider scholarship on norm adoption that forms the theoretical backdrop for this study. We then describe the study’s methodology. Thereafter we present a historical narrative on the political dynamics of adoption and implementation of sexuality education policy in Nigeria. We organize the narrative by phases of the policy process: agenda-setting, formulation, implementation and evaluation. In the discussion, we identify the factors that have shaped the Nigerian policy trajectory, drawing on theory pertaining to international norm diffusion and adoption. We argue that this body of theory and the debates it has stimulated are useful for understanding policy dynamics for a variety of health and population issues that low and middle-income countries face. We also identify principles that emerge from the Nigerian case relevant for managing the politics of sexuality education in other socially conservative settings.

### Theoretical backdrop

The influence of international norms on national policies is a central concern in international relations and sociology scholarship. Norms are collective expectations for proper behavior among actors with a given identity [14]. They apply to governments, among other actors, and to many kinds of issues, from human rights to education to warfare. For instance, most governments accept the norm that non-combatants in warfare be treated as neutral. Scholars differ on several issues pertaining to international norms and their power to shape national behavior. These include (1) whether they shape national behavior in any meaningful way, particularly vis-à-vis national interests; (2) the role of human agency versus structure in shaping their adoption; and (3) their power to alter national and local norms.

These debates are directly relevant to understanding the political dynamics of sexuality education policy in low-resource settings. Do those governments that adopt policy do so based primarily on judgments of appropriateness (for instance, the idea that adolescents have a right to information that will facilitate their empowerment) or on assessment of national interests (for instance, an appraisal that sexuality education will help avert potential social instability posed by HIV/AIDS)? And is the cross-national spread of school-based sexuality education a product primarily of advocacy by champions, or of the diffusion of a global culture that privileges rights and rationality?

The interests versus norms debate has pitted rationalists against constructivists. Rationalism in the form of neorealist theory views the international system as anarchic: a self-help world with states pursuing security and power above all else [13]. Constructivists [15, 16] challenge rationalists, arguing that self-interest—or the logic of consequences—is not the sole driver of state behavior, and that ideas, not just material resources, shape outcomes. States—and other actors in the international system such as international non-governmental organizations that rationalists pay little attention to—operate also on a logic of appropriateness, a sense of what ought to exist. The cross-national spread of women’s suffrage is one example of the power of norms [17].

The structure versus agency debate has taken various strands in international relations scholarship, and has been heavily influenced by sociological treatment of this subject (see for instance [18]). Among some constructivists, it has manifested as a debate between sociological institutionalists and those who advance an agentic-oriented constructivism [19]. Sociological institutionalists note the remarkable homogeneity in the structure and aspirations of states that, they argue, cannot be explained without appeal to the power of a world culture, one that privileges rights and modernity [11]. The enactors of the policies, programs, and bureaucratic structures that bear such similarity across diverse states are not so much engaged in careful deliberation as in enacting accepted scripts. Agentic-oriented constructivists criticize the mechanistic thrust of sociological institutionalism, seeing contingency in the world, and the role of human deliberation in change.

The international-local debate concerns the power of international norms to alter local belief systems [20]. Several constructivist works ascribe considerable power to international norms. For instance, Keck and Sikkink [21] provide evidence of the influence of transnational advocacy networks—advancing principled ideas pertaining to human rights and the environment, among other issues—on national policies. Yet scholarship in this vein has been criticized for presenting governments as ‘norm-takers,’ for presuming the flow of norms is largely global to local, for downplaying the considerable local resistance to international norms, for overlooking the local origins of many international norms and for missing the considerable heterogeneity of norms that exists at local levels [12, 22, 23].

While few scholars take absolute positions in these debates surrounding international norms, many have distinct standpoints, emphasizing the power of interests rather than norms, structure rather than agency, international rather than local influences—or vice-versa. Some scholars have reacted to these strong positions by seeking ways to bridge differences. Sil and Katzenstein [24], for instance, call for abandoning paradigm-bound research in favor of considering multiple perspectives—‘analytical eclecticism’ in the study of international relations—drawing on insights from each of the major paradigms and recognizing synergies among them. Wendt [25] argues for developing theories grounded in structuration—the mutual constitution of structure and agency—rather than ones that have an exclusively structural or individualistic ontological foundation. Finnemore and Sikkink [17], although recognized as constructivists, reject the interest-norm divide as simplistic, advancing the idea that much social change can be understood via processes of strategic social construction—actors instrumentally pursuing principled concerns to alter social reality. Principled concerns, they argue, *constitute* the interests of some actors.

Sexuality education in Nigeria represents, prima facie, a hard case for theories claiming strong influence of international norms on national policy-making. Conservative social values are widespread in Nigeria, and sexuality education touches on highly sensitive issues, including adolescent sexuality and marriage. For these reasons, we might expect international initiatives to have little influence on national policy. On the other hand, particularly since the 1994 International Conference on Population and Development (ICPD) in Cairo, initiatives to advance reproductive health in low-income countries have gathered momentum, putting pressure on national governments to move on these issues. There are reasons, therefore, both to question and to expect international normative influence in this case.

In the sections that follow, we examine the historical trajectory of sexuality education in Nigeria, considering the case with reference to these arguments. We ask why Nigeria adopted a sexuality education curriculum when social norms mitigated against school-based delivery. In doing so, we consider whether the origin of sexuality education in Nigeria is best understood in terms of national interests, norms, or some mix; if policy evolution was primarily a product of structural forces, agentic, or interactions between the two; and whether the forces that have shaped sexuality education in the country were predominantly global, local, or an amalgam.

## Methods

This is a historical case study, conducted with the aim of understanding the evolution of sexuality education policy in Nigeria. We selected a case study methodology because it considers a phenomenon in its real-life context, thereby giving it the capacity to reveal underlying causal mechanisms and processes [26].

In 2014 we conducted 52 interviews (Appendix) with people from Nigerian non-governmental organizations (including ones based in Lagos, Oyo, Niger, Kano and Abuja), the federal government (including the Ministry of Education, the Ministry of Health and the National Agency for the Control of AIDS), international foundations (including Ford and MacArthur), international organizations, state governments, religious institutions, universities and bilateral donors. We identified these individuals through publicly available documents and consultation with persons working on the issue. Most of the interviews were conducted in person in five locales in Nigeria—Lagos, Abuja, Ibadan, Minna and Kano. Most interviewees had been involved in promoting sexuality education in the country; others were critics of sexuality education; still others were impartial observers of these processes. We adopted a purposive rather than sampling selection strategy: the aim was not to obtain a representative sample but to reach key actors, critics and observers and to achieve theoretical saturation—the point at which no major new concepts or information would be gleaned by additional interviews. We developed a semi-structured interview instrument with mostly open-ended questions. Although we asked some questions of most interviewees, we tailored the selection of questions to each interviewee to elicit his or her unique knowledge. We informed each interviewee that responses would be kept confidential, but that we might excerpt some quotations for inclusion in the article. Given the sensitive nature of this topic we did not record interviews but instead took detailed notes. We conducted interviews one by one, most with two or three of the investigators present. One person took detailed notes; after the interview the others who had been present added to the note-taker’s notes, and any discrepancies were discussed. If discrepancies could not be resolved, we contacted the respondent again. The average interview length was one hour.

In addition, we gathered and analyzed more than 300 documents pertaining to sexuality education in Nigeria and globally (Table 1). They were of the following types: research on sexuality education programs in Nigeria; research on sexuality education programs across the world; Nigerian non-governmental organization (NGO) documents; international donor, international organization and Nigerian government reports; research and information on the Nigerian social, political and economic context; Nigerian demographic and health surveys; and documents concerning global sexuality education policy. We gathered these through several mechanisms. First, we searched online databases, including Google Scholar, ProQuest, JSTOR, Medline and Popline, using some combination of the keywords ‘Nigeria’, ‘sex education,’ ‘sexuality education,’ ‘HIV/AIDS’, ‘family planning’, ‘adolescent’, ‘Kano’, ‘Niger’ and ‘Lagos.’ We searched for documents from 1960 to the present. Second, we searched for articles in major journals that publish on international family planning and reproductive health, including *Studies in Family Planning, Social Science and Medicine, Health Policy and Planning, African Journal of Reproductive Health* and *International Perspectives on Sexual and Reproductive Health.* Third, we asked our key informants for documentation pertaining to sexuality education policy processes in Nigeria. They possessed some of these documents and referred us to others, which we secured through online searches. Fourth, we gathered documents from a resource center on young people’s sexual and reproductive health, maintained by Action Health Incorporated (http://www.actionhealthinc.org/publication/). We visited the resource center in Lagos, Nigeria and gathered hard copies of documents there. We also downloaded available online documents from that center. Finally, we collected newspaper and media reports via ProQuest Newsstand, as well as through specific national and local newspaper online archives.

**Table 1:** Documents analyzed

Document type	Percentage of total documents	Example
Research on sexuality education programs in Nigeria	40%	Ibadan Social and Evaluation Research Team (ISERT). Evaluating the Implementation of Sexuality and Life Skills Education among In-School and Out-of-School Adolescents in Nigeria. Lagos, Nigeria: Ford Foundation; 2014.
Research on sexuality education programs across the world	30%	Haberland N. The case for addressing gender and power in sexuality and HIV education: A comprehensive review of evaluation studies. Int Perspect Sex Reprod Health. 2015;41:31–42.
Nigerian NGO documents	13%	Action Health Incorporated (AHI). The AHI Story 1989–2001. Lagos. Nigeria: Action Health Incorporated; 2002.
International donor, international organization and Nigerian government reports	7%	United Nations Population Fund (UNFPA). State of the World Population 2013: Motherhood in Childhood Facing the Challenge of Adolescent Pregnancy. New York: UNFPA; 2013.
Research and information on Nigerian context	5%	Bergstrom K. Legacies of Colonialism and Islam for Hausa Women: An Historical Analysis, 1804–1960. Michigan State University Working paper # 276. East Lansing, Michigan, USA: Michigan State University; 2002.
Nigerian demographic and health surveys	2%	National Population Commission (NPC) [Nigeria], ICF International. Nigeria Demographic and Health Survey 2013. Abuja, Nigeria and Rockville, Maryland, USA: NPC and ICF International; 2014.
Documents on global sexuality education policy	2%	SIECUS. Guidelines for Comprehensive Sexuality Education: Kindergarten-12th Grade. 1st ed. New York: SIECUS; 1991.

We analyzed interview notes and documents to piece together a historical narrative of the development of Nigerian sexuality education policy. We coded and excerpted relevant information into a document, organizing it chronologically and by potential causal factors shaping policy dynamics. We adopted a process-tracing methodology [27], examining evidence surrounding alternative explanations and seeking to detect the mechanisms linking presumed causal factors with the policy outcomes of interest.

To minimize bias, we employed several recommended techniques [26, 28]. First, we triangulated among sources. Our information came not just from interviews but also from published sources and independent reports. Second, to avoid recall bias, we relied on sources beyond individual interviews to check historical accuracy. We also inquired about events with multiple respondents. In several instances, historical information offered in different interviews was inconsistent or diverged from that contained in written sources. In those cases, we relied on published, peer-reviewed written works as the most credible source of information. Finally, we solicited and incorporated feedback on drafts of the paper from four individuals familiar with the history of efforts to address sexuality education.

One limitation of this study is its focus on just one country. This feature constrains us from drawing broader inferences on the power of international norms and on factors driving sexuality education programs in other contexts. We are therefore cautious in making claims, recognizing that considerably more research on the politics of sexuality education in other settings is necessary to assess the generalizability of our findings. Another limitation is the fact that the funder of this study, the John D. and Catherine T. MacArthur Foundation, has promoted sexuality education in Nigeria, potentially introducing bias in our interpretation of findings. We sought to guard against such bias by (1) relying on sources beyond those provided by the Foundation to document its role in the history of sexuality education in Nigeria; and (2) securing the Foundation’s agreement that this study constitutes independent research, and that the authors have complete control in the reporting of results.

## Results

The history of school-based sexuality education in Nigeria can be divided into three phases outlined in Table 2. During an agenda-setting phase from 1989 to 1996, domestic champions, aware of the problems Nigerian adolescents faced and of global initiatives on reproductive health, and with the support of international donors, founded NGOs. They worked through these NGOs to generate national attention on the need for sexuality education. The policy formulation and adoption phase took place between 1997 and 2002. During that time, these champions, with international donor support and allies inside government, advanced domestic sexuality education initiatives leading to the national adoption of policy on sexuality education. Objections subsequently compelled dilution of its content. Finally, the implementation and evaluation phase proceeded from 2003. During this time, several states have advanced considerably in delivering sexuality education to students; many others have made little headway. Central to policy development have been domestic NGO-international donor alliances pushing Nigerian federal and state governments to act.

**Table 2:** Key developments in history of sexuality education in Nigeria

Phase 1 *Agenda-setting* *1989-1996*	• National Adolescent Health Policy (1995) • Task force to adapt US sexuality education guidelines to Nigerian context (1995) • Guidelines for Comprehensive Sexuality Education in Nigeria (1996)
Phase 2 *Policy formulation and adoption* *1997-2002*	• Government concern about HIV epidemic grows • National Strategic Framework for the Implementation of Adolescent Reproductive Health Programs in Nigeria (1999) • Nigeria transitions to democracy (1999) • Passage of ‘National Comprehensive Sexuality Education Curriculum’ (2001) • Curriculum changed to abstinence-only, renamed ‘Family Life and HIV Education’ (2002)
Phase 3 *Implementation and evaluation* *2003-onwards*	• Implementation of curriculum varies greatly from state to state • Global Fund to Fight AIDS, Tuberculosis and Malaria provides funding for curriculum implementation (2010) • Global Fund reduces number of states receiving funding to implement curriculum (2013)

Note: Adapted from Table 1 [64]

### Phase 1, agenda-setting: 1989 to 1996

#### The emergence of domestic champions

As early as the 1970s in Nigeria, sexuality education was included in teacher training curricula, but many teachers were embarrassed to implement it, and it often fell by the wayside [29]. During the 1980s, as better demographic data became available and international donors pressed for reductions in population growth, Nigeria implemented a limited program on population and family life education, although it was carried out only in a few schools [30, 31]. Prior to the 1990s, two reproductive health NGOs—Planned Parenthood Federation of Nigeria and the Society for Family Health—conducted outreach on sexuality education topics, but in-school youth were not their primary targets [32]. It was not until the 1990s that consolidated action around sexuality education emerged.

Recognition by domestic champions of the dangers Nigerian adolescents faced from pregnancy, HIV and other issues connected to sexuality and reproduction sparked the process leading to national adoption of school-based sexuality education. One of these champions recalls her own school years (Interview (I) 8):

I attended a boarding school which was co-educational. We had pit toilets. And in the toilets, I saw fetuses, although I never knew then they were fetuses. Nobody spoke to you about getting pregnant.

These formative experiences and subsequent observations of the adverse conditions Nigerian adolescents faced, led two of these individuals to establish Action Health Incorporated (AHI) in 1989, an NGO whose mission is to improve the health of Nigerian adolescents. A major break for the organization came in 1992, when AHI secured a grant from the MacArthur Foundation for a three-year work program on adolescent reproductive health [33]. MacArthur representatives had paid a visit to one of AHI’s founders and encouraged her to write a proposal, a skill she had learned through prior work at another Nigerian NGO (I8).

#### The development of national guidelines on sexuality education

The connection of AHI’s co-founder to the MacArthur Foundation facilitated a crucial encounter that shaped subsequent developments for sexuality education in the country. In 1992, she traveled to Cuernavaca, Mexico, where the Foundation had organized a small meeting of grantees (I8; I22) [34, 35]. There she heard the deputy director of the Sexuality Information and Education Council of the United States (SIECUS) present on guidelines the organization had developed the previous year for sexuality education for the United States [36]. She met with the deputy director and concluded that Nigeria needed the same.

She subsequently initiated a process that led to the development of Nigerian guidelines [34, 35]. With funding and technical support from SIECUS, and additional funding from the MacArthur Foundation, she organized the National Guidelines Task Force in 1995, which included NGOs, medical associations, UN agencies, officials from the Federal Ministries of Education and Health, researchers, media and religious representatives. Many had been working independently on adolescent reproductive health. This was the first explicit effort in the country to build a policy community surrounding sexuality education.

The 1994 ICPD facilitated her efforts by providing domestic legitimacy for this agenda. Attended by a sizeable contingent from Nigeria, the ICPD was a watershed, helping to reshape global norms away from population control and toward the right of individuals to make their own choices surrounding reproduction [37]. The government of Nigeria signed the Program of Action, an entire section of which concerned adolescent health.

In 1995 the Task Force revised the SIECUS guidelines to make them appropriate for the Nigerian context, in the process considering controversial subjects such as abortion and sexual orientation [35]. On October 1996, the Minister of State for Education publicly released the Guidelines (I8) [34], endorsed since by more than 100 organizations [35].

#### The appearance of a state-level prototype program

In a simultaneous but separate process, another Nigerian NGO moved the country toward national adoption of sexuality education. The Ibadan-based Association for Reproductive and Family Health (ARFH) was founded in the same year as AHI (1989). Its Life Planning Education program began in Ibadan in 1994 in response to concerns about rising incidence of unwanted pregnancy and adolescents dying from septic complications of abortion (I21) [38]. ARFH secured funding from the United Kingdom’s development agency to scale-up the program in Oyo state, partnering with the state government to do so (I21). With support from the United Kingdom and the Ford Foundation, from 2004 on the program was introduced into five other states: Gombe, Bauchi, Borno, Yobe and Kebbi (I21) [38]. For more than a decade, ARFH was also involved in a program training members of the National Youth Service Corps—a Nigerian government-organized year of service for all university graduates—to provide education to peers on sexuality education (I21).

Other NGOs also participated in the coalition that drafted the Guidelines and developed state-level programs [31]. Among the most active were Girls’ Power Initiative (GPI) in Cross River state and the Adolescent Health and Information Projects (AHIP) in Kano state. Many of these NGOs were beneficiaries of a MacArthur Foundation program designed to foster leaders for reproductive health [39].

### Phase 2, policy formulation and adoption: 1997 to 2002

The creation of the National Guidelines on Sexuality Education set the stage for the Federal Government’s forward movement on sexuality education, including the adoption of the Guidelines and their subsequent implementation in schools. Several national and international developments shaped this decision: rising national concern over HIV/AIDS; the entry of the Ford Foundation into adolescent sexual and reproductive health in Nigeria; and, especially, advocacy by domestic champions.

#### A national conference on adolescent reproductive health

Over the period 1986 to 2001, prevalence of HIV in Nigeria rose to 5.8% [40]. The national policy response was lethargic until 1997, when former Minister of Health Olikoye Ransome-Kuti, a widely respected physician sympathetic to prioritizing reproductive health, shocked the nation by announcing that his brother Fela, a famous musician, had died of AIDS-related complications [32] (I17; I19; I20). In the view of several sexuality education proponents, it was AIDS, more so than teen pregnancy, that opened the national conversation about national sexuality education (I9), in part because HIV affected boys as well as girls. As one put it (I8):

Teenage pregnancy was a major issue but we had not gone far before HIV came into the picture... HIV now necessitated talk about sexuality. If a girl dies, it’s not an issue. But if a boy dies, yes.

Beginning in 1997, Ford Foundation support facilitated the process of national policy adoption for sexuality education in Nigeria. It made available additional resources and technical expertise to domestic NGOs, including AHI in Lagos, ARFH in Ibadan, AHIP in Kano, GPI in Calabar, Inter-gender in Jos and Life Vanguards in Osun.

With the availability of new resources, the existence of the National Guidelines on Sexuality Education, growing concern over HIV/AIDS, and the five-year anniversary of ICPD on the horizon, domestic champions began strategizing on how to further the cause of adolescent reproductive health and to bring coordination to disparate initiatives across the country. They hit upon the idea of a national conference on adolescent sexuality and reproductive health (I8), which would also serve to operationalize the government’s commitments from the 1995 National Adolescent Health Policy [41]. Drawing on resources from several international agencies—particularly from a Ford Foundation grant [42]—AHI joined with a core group of champions from organizations working on adolescent reproductive health to organize this conference (I2; I9). The group was diverse, including individuals from foundations (Ford, MacArthur); domestic NGOs (AHI, GPI, AHIP); the government (the Federal Ministry of Health); and United Nations agencies (UNICEF, UNFPA) (I9). To secure the involvement of the Government, AHI handed over official leadership of the process to the Ministry of Health [42].

The conference was held at the Sheraton in Abuja in January 1999 [42, 43] (I9). In attendance was the Minister of Health (I9). At the conference, delegates came to an agreement that sexuality education should be included in a national school curriculum [42]. Toward that end, they approved the National Strategic Framework for the Implementation of Adolescent Reproductive Health Programs in Nigeria [42]. One official described the significance of the conference as, “the first place at which the semblance of a national coalition formed” (I9). Another respondent attributed the straightforward approval of the Strategic Framework to the military regime, which was otherwise occupied at the time (I2).

#### The national adoption of a sexuality education curriculum

Aside from these public efforts, the AHI founders also sought adoption of a curriculum throughout the country (I2; I8). Toward that end, they approached an official in the Ministry of Education who supported its policy-making bodies. The official immediately took to their ideas on sexuality education, noting that (I4):

The problem of HIV was there but the Ministry did not know where to start from in terms of talking about sex vis-à-vis HIV/AIDS.

The official advised the AHI founders to prepare a memorandum, which the official then herself presented to education officials. While some education commissioners from more conservative northern states expressed concerns that such a curriculum would corrupt girls, the official and her superiors in the ministry worked hard to convince them of its value. She assured them that it was not about sex, but rather about its prevention (I4):

Teaching our girls about their bodies and how to say no to immorality…how to handle those who would want to spoil them, and not to succumb to pressure to anybody who wants to pollute you.

At a 1999 meeting of the National Council on Education, the Ministry’s highest policy-making body, education officials from each state approved a proposal for including sexuality education in schools (I4) [34, 44]. Thereafter the proposal was passed on to another body in the Ministry, a parastatal responsible for school curriculum development—the Nigerian Educational Research and Development Council (NERDC) (I4). In collaboration with several state institutions and AHI, NERDC developed the curriculum content [31, 34, 45, 46]. AHI secured financing from the International Women’s Health Coalition and the Packard Foundation to support this process (I9) [34].

At its 48th session in Enugu in August 2001, the National Council on Education formally approved the curriculum for use in Nigerian schools [34, 43, 47]. The curriculum’s content was comprehensive and included information on contraception, sexual abuse, gender roles, female genital mutilation, sexual orientation, masturbation, and abortion, among other subjects [34, 48].

#### Controversy and dilution of the curriculum

Not long after approval, resistance to the curriculum emerged (I2; I8; I9) [31, 48]. In July 2002, a prominent national newspaper, *The Weekly Trust*, after talking to Islamic clerics and the Anglican Archbishop of Kaduna, published an editorial highly critical of the curriculum, claiming it promoted immorality throughout the country (I27). Shortly thereafter, at a meeting in 2002 in Kaduna involving Federal Ministry of Education officials and State Commissioners of Education, the curriculum came under threat. Most of the commissioners from the more conservative northern states objected to the idea of such a curriculum and to its content (I2; I8). Alerted of this possibility, the AHI founders traveled to the meeting, where, behind the scenes, sympathetic ministry officials kept them apprised and consulted them on negotiations. As they noted (I8):

We had prior information that the northern governments had agreed they would not have the document so we tried to get the middle-belt states out of the clutches of the core northern states. The southern states were on our side.

Eventually ministry officials and the commissioners reached a compromise, but one that significantly diluted the content of the curriculum to abstinence-only [48]. Mention of contraception, masturbation, abortion, sexual diversity and other contentious topics was removed, as was the word ‘sexuality.’ States could tailor content to conform with local socio-cultural sensitivities. Students would not be tested on content. In addition, the title was changed from ‘Sexuality Education Curriculum’ to ‘Family Life and HIV/AIDS Education,’ now known by its acronym, FLHE. Proponents reluctantly agreed to these modifications, realizing these were the only way to get a majority of commissioners to approve the curriculum.

### Phase 3, implementation and evaluation: 2003-onwards

Following national government approval of the curriculum, proponents sought to ensure its implementation across the country. They have had variable success. In Lagos, Oyo and several other states, most junior secondary students (mostly in the 11–16 age range) received sexuality education. In many other states, few students are exposed to the curriculum.

#### Major developments on implementation in Nigeria

Implementation proceeded slowly after adoption of the curriculum. Seven years after being instructed to do so, nearly a quarter of Nigeria’s 36 states had yet to introduce the curriculum [31]. A 2008 study noted limited awareness of policies at state and local government levels, low levels of political commitment and minimal budgets to carry out the program [49]. In response, the Federal Ministry of Education in 2008 issued guidelines for FLHE implementation [50]. In addition, in a move that helped to institutionalize FLHE in the schools, the National Commission for Colleges of Education (NCCE) made it mandatory for first year students in national colleges of education to take a course entitled ‘Family Life and Emerging Health Issues’ that covered (and even went beyond) the FLHE curriculum [51].

In 2010, a new attempt was made to implement the curriculum on a national scale. In that year the Global Fund to Fight AIDS, Tuberculosis and Malaria approved a grant to ARFH to support FLHE implementation across Nigeria [52]. The Federal government was ARFH’s grant sub-recipient. The original goal was to train nearly 50,000 teachers over the course of five years during two grant phases. To initiate the project ARFH and government officials visited all 36 states and the Federal Capital Territory, setting up project advisory committees in each composed of political, traditional and religious leaders (I21). A second phase of the Global Fund program began in 2013, with another NGO—the Society for Family Health—as grantee and the Federal Ministry of Education as sub-recipient (I28). The Global Fund support spurred, among other effects, the release of funds for FLHE by some states, augmented teacher training and the production of teacher handbooks and curriculum guides (I21).

#### Implementation effectiveness

Implementation results have varied by state. Implementation in Lagos has proceeded further than in most. All the state’s 320 public junior secondary schools have taught the curriculum [53]. A UNESCO study estimated that the schools reached 716,000 students cumulatively between 1999 and 2009 at a cost of only $6.90 per pupil [54]. A 2009 evaluation of the program’s effectiveness found that students exposed to the program demonstrated a large increase in knowledge of sexuality and HIV, and support for abstinence and gender role equality [55]. The state’s cosmopolitan cultural environment facilitated implementation: 89.3% of women between the ages of 15 and 49 are literate, for instance, compared to 53.1% nationally [56]. Initiative by the Lagos-headquartered AHI and the state government, drawing on resources from the MacArthur and Ford Foundations among other institutions, facilitated effective implementation. Yet even in Lagos the program has not been immune to controversy or critique. In 2005 an NGO criticized it for promoting immorality among children, accusing it of pushing students to study techniques of, “masturbation, caressing, withdrawal, ejaculation, erection, [and] taking of drugs among others to avoid pregnancy” [57].

Implementation in Oyo state has also been relatively effective compared to other states, largely due to the ARFH-led Life Planning Education program. ARFH partnered with the state government to carry out the program [38], securing funding from the United Kingdom—which lasted through 2003—to do so. Between 1999 and 2003, the program expanded to 131 of the state’s 324 public secondary schools, including at least one school in each of the state’s 33 local government areas, and benefited about 425,000 young people [38]. The World Bank reported significant program effects, including a decline in unplanned pregnancies and abortions [38]. The program’s success led to its replication in five other states from 2004 on, with support from the Ford Foundation [38] (I21).

Kano presents a contrast to Lagos and Oyo. Despite donor funding to the effective local NGO, AHIP, that has consistently championed the cause, efforts to scale-up FLHE have encountered considerable resistance (I44; I45). Local champions for FLHE have found themselves in life-threatening situations. A Ministry of Education official estimates that the proportion of schools covered may be as low as 10% (I44). One reason for the low coverage is that Kano’s social and cultural environment is much less conducive to promoting sexuality education than that of Lagos and Oyo. Conservative forms of Islam have been central to the social and political life of the people for more than two centuries [58, 59], and only 36.3% of women between the ages of 15 and 49 are literate [56]. Still, local champions have made some headway. With funding from the MacArthur Foundation, since 2008 they have carried out a modified version of the FLHE curriculum in some of the state’s 4000 Islamiyya Schools—modern Quranic schools that incorporate secular education.

Implementation has also been variable across the rest of the country. A study by researchers at the University of Ibadan found that the percentage of secondary schools within states implementing FLHE ranged from 13.5 to 100%, and most states have no budget line for the program [31]. A study on program implementation [60] found multiple challenges, including a limited number of trained teachers, leading to the curriculum being delivered by untrained teachers; crowded classrooms; insufficient learning materials; and inadequate monitoring mechanisms.

## Discussion

Scholarship concerning the influence of international norms on national policy-making helps to identify factors that shaped the adoption and implementation of sexuality education policy in Nigeria. Debates in this body of work pertain to the relative influence of norms and interests, agentic and structural forces, and local and global factors in shaping national policy processes. Much of this scholarship emphasizes the influence of one or the other element of these binaries. The Nigerian case, however, reveals interactions among rather than the dominance of any particular element.

Norms shaped policy adoption: government officials came to accept the appropriateness of the idea that adolescents should have information on sexuality, and responded by developing and approving national sexuality education curricula and programs. However, interests also influenced their decisions: the national government prioritized sexuality education only when officials became alarmed about the spread of HIV. Moreover, sexuality education advanced not only on the power of ideas, but also on the availability of material resources: the willingness of international donors, and a few Nigerian state governments, to provide funding.

Agency mattered: moved by their concern about the reproductive risks that Nigerian adolescents faced, domestic champions promoted the cause of sexuality education. This entrepreneurship may be the single most powerful factor behind the advance of the agenda. However, these champions did not develop their ideas in isolation. They participated in global gatherings such as the ICPD, interacted in international meetings with donors, and joined global movements for sexual and reproductive health and rights. These champions were shaped by a world culture they did not create—an international social structure that privileges rights and modernity and that produced the conditions for the emergence of global initiatives on reproductive rights.

Local and national norms shaped the policy process: in places such as Kano, the beliefs that school-based sexuality education is a Western imposition and fosters adolescent sexual experimentation posed considerable barriers to implementation. In addition, suspicion by northern state commissioners of education about sexuality education led to dilution of the national content of the curriculum, resulting in an abstinence-only program. However, international norms also heavily influenced policy development. Most notably, the very idea of school-based sexuality education came from abroad.

Findings from the Nigerian case are in line with claims and approaches that emphasize theoretical integration. Sil’s and Katzenstein’s [24] appeal for analytical eclecticism—drawing on multiple paradigms for insights—is relevant. Both constructivism, which emphasizes norms, and rationalism, which emphasizes interests and material influences, direct us to factors that help to explain why the Nigerian government came to embrace sexuality education. Finnemore’s and Sikkink’s concept of strategic social construction [17] is also relevant. NGOs acted deliberately to alter social reality: the advance of sexuality education, a principled concern, came to *constitute* their interests.

Wendt’s ideas on structuration [25] that integrate agency and structure are also pertinent. An international social structure—a world culture privileging rights and modernity—was generative of the national and local champions who became the strongest proponents of sexuality education [19]. They did not come up with their normative beliefs or ideas about the efficacy of human action independent of the social forces that surrounded them. Yet they were not merely enacting scripts; they engaged in careful deliberation, motivated to act on real problems they witnessed in society. And in choosing to act, they initiated a process that may ultimately lead to broad shifts in the set of inter-subjectively held beliefs that political and social leaders in the country hold on sexuality education—a potential change in social structure.

Integrative analytical approaches undoubtedly are relevant and worth employing for understanding the influence of international norms on many health and population policy areas, including refugees, HIV/AIDS and family planning. For instance, norms and interests intersect in refugee policy: a humanitarian imperative to protect populations at risk confronting national concerns over identity and security. They do so as well in HIV/AIDS policy: the norm of right to treatment, the stigmatization of affected groups, and the fear by governments of cross-national contagion and social disruption all shape national policy responses. Agentic and structural forces help to explain the diffusion of policy on family planning: for instance, the power of national champions in places such as Indonesia, Bangladesh and Iran, all participating in a world culture advancing the idea of fertility control as an element of modernity.

## Conclusion

### Implications for future research on international norms and national policy-making

We offer three ideas on how integrative approaches from scholarship on international norms might be applied usefully to the analysis of health and population policy—one idea drawn from each of the three large debates. First is the value of attentiveness, when conducting health and population policy analysis, to forces both internal and external to countries. In political science scholarship, comparativists have typically focused on internal forces. One consequence, as Finnemore [15] and Meyer and colleagues [11] have pointed out, is to overlook the remarkable cross-national homogeneity that exists in the timing and the nature of adoption of policy on particular issues—evidence that forces external to the country are at work. The role of international actors in the spread of national population stabilization policies post-World War Two is an example [61, 62]. International relations scholars, by contrast, often have concentrated on global policy dynamics to the neglect of the national. The danger in doing so, as Elgström [22] and Zwingel [12] have noted, is the disregard of local and national forces that are sources of policy adoption as well as mechanisms of subversion of and resistance to international pressures.

Second is to adopt both long and short-term perspectives on health and population policy change. Kim and Sharman [19], drawing on Pierson [63], critique agentic-oriented constructivists for focusing excessively on the role of norm entrepreneurs, arguing that they attribute too much power to the efforts of individuals and are too short-term in orientation. Pierson, calling for a longer-term structural perspective, argues that (p. 178):

Many important social processes take a long time…to unfold…, [which is] problematic…in areas of inquiry where individual strategic action has become the central vantage point for framing questions and answers about social life.

On this point, one might consider patterns in national policy responses to the HIV/AIDS pandemic, best understood over the course of decades rather than at any given moment in time. Yet Pierson himself, in making this critique, also points to the opposite difficulty: an “overly deterministic picture of social processes” (p. 185). Detecting contingency requires attention to agency. Moreover, this agency may have long-lasting effects. A structurationist approach [25] that emphasizes the mutual constitution of structure and agency, rather than one grounded in a pure structuralist or individualist ontology, offers the most promise for understanding health and population policy development.

Third is the idea that in investigating the motivations of political and social actors surrounding health and population policy, it may be worth setting aside, at least temporarily, the impulse to label these quickly as grounded either in ‘norms’ or ‘interests.’ In explaining the same act—say a head of state’s public support for or condemnation of legalized abortion—a constructivist may be inclined to identify it as motivated by a principle-based concern; a rationalist to point to the interest that drives it. Yet the motivations may be multiple, intertwined and unclear even to the actor him or herself. The analyst might best direct his or her effort to detecting the complex motivations at work without prejudging which category these fall under. Finnemore and Sikkink argue in their 1998 piece [17] that, “Instead of opposing instrumental rationality and social construction we need to find some way to link those processes theoretically” (p. 910). The history of health and population policy in low and middle-income countries offers scholars rich empirical evidence to deepen our understanding of how social construction and rationality may interact to shape policy diffusion, adoption and implementation.

### Implications for future research on sexuality education in Nigeria and elsewhere

Aside from implications for scholarship on the influence of international norms on national policy-making, the case study also points to several principles on managing the politics of sexuality education in socially conservative settings. Our knowledge of how to manage these politics would benefit considerably by comparative studies that consider and critically examine these principles. Among these principles are [64, 65]:

- *Understand that national proponents are the core actors*. External actors including international donors and United Nations agencies may offer financial and technical resources needed to support the issue. However, without local champions committed to the issue, knowledgeable about the social context and savvy about how to negotiate the political environment, sexuality education may not advance far.
- *Anticipate opposition.* Even in the most favorable social environments, some opposition to school-based sexuality education is to be expected as it gives rise to fears that adolescents may be encouraged to engage in sexual experimentation. Presumably, proponents will be most effective when they anticipate the emergence of such opposition, actively seek to understand the objections, and identify strategies to address these concerns and neutralize opposition.
- *Take advantage of policy windows.* Political opportunities to promote sexuality education arise unexpectedly. For instance, the alarm caused by the spread of HIV/AIDS in Nigeria presented an opportunity for proponents to advance sexuality education as part of the solution. Proponents are more likely to make progress on the issue when they are attentive to these political opportunities and are ready to act when these windows appear.
- *Recognize sub-national variation.* Contexts vary not just nationally but sub-nationally, shaping prospects for implementation. In Nigeria, for instance, the conservative social context in Kano State was much less conducive to sexuality education promotion than that of more cosmopolitan Lagos—impeding progress in the former. Proponents need to consider this sub-national contextual variation and adapt strategies accordingly.

While the case of sexuality education in Nigeria is unique, these findings indicate its relevance to broader questions on the influence of international norms on national policy-making—specifically the value of adopting integrative rather than binary perspectives. The case study also suggests strategies likely to be helpful to proponents of sexuality education working in other socially conservative contexts.

## Appendix

**Table 3:** List of interviews

Interview	Location	Interview number	Gender
NGO representative, 26 May 2014	Lagos, Nigeria	1	F
NGO representative, 26 May 2014	Lagos, Nigeria	2	M
Former donor official, 26 May 2014	Lagos, Nigeria	3	M
Former government official, 28 May 2014	Lagos, Nigeria	4	F
Government officials, 28 May 2014	Lagos, Nigeria	5	M and F
Researcher, 29 May 2014	Lagos, Nigeria	6	M
NGO representative, 30 May 2014	Lagos, Nigeria	7	M
NGO representative, 30 May 2014	Lagos, Nigeria	8	F
Former donor official, 2 June 2014	Lagos, Nigeria	9	M
Civil society representative, 2 June 2014	Lagos, Nigeria	10	F
Former government official, 3 June 2014	Lagos, Nigeria	11	F
Educator, 3 June 2014	Lagos, Nigeria	12	F
NGO representative, 3 June 2014	Lagos, Nigeria	13	F
School official, 4 June 2014	Lagos, Nigeria	14	M
Educators, 4 June 2014	Lagos, Nigeria	15	F
School official, 4 June 2014	Lagos, Nigeria	16	F
Former government official, 4 June 2014	Lagos, Nigeria	17	F
Former government official, 5 June 2014	Lagos, Nigeria	18	F
Former donor official, 5 June 2014	Lagos, Nigeria	19	M
NGO representative, 6 June 2014	Ibadan, Nigeria	20	M
NGO representative, 6 June 2014	Ibadan, Nigeria	21	M
NGO representative, 7 June 2014	Lagos, Nigeria	22	M
NGO representative, 7 June 2014	Lagos, Nigeria	23	F
Researcher, 6 October 2014	Abuja, Nigeria	24	M
Former government official, 7 October 2014	Abuja, Nigeria	25	M
Government officials, 7 October 2014	Abuja, Nigeria	26	F
Donor official, 7 October 2014	Abuja, Nigeria	27	M
Former government official, 7 October 2014	Abuja, Nigeria	28	F
NGO representative, 8 October 2014	Abuja, Nigeria	29	M
NGO representatives, 9 October 2014	Abuja, Nigeria	30	M and F
International NGO official, 9 October 2014	Abuja, Nigeria	31	F
International NGO official, 10 October 2014	Abuja, Nigeria	32	M
Government official, 12 October 2014	Abuja, Nigeria	33	F
Government official, 13 October 2014	Abuja, Nigeria	34	M
Former government official, 14 October 2014	Minna, Nigeria	35	M
Former student, 14 October 2014	Minna, Nigeria	36	F
UN Agency representative, 14 October 2014	Minna, Nigeria	37	F
NGO representative, 15 October 2014	Minna, Nigeria	38	M
NGO representative, 15 October 2014	Minna, Nigeria	39	M
State government official, 15 October 2014	Minna, Nigeria	40	M
Former NGO representative, 15 October 2014	Minna, Nigeria	41	M
State government official, 16 October 2014	Minna, Nigeria	42	M
NGO representative, 16 October, 2014	Minna, Nigeria	43	F
State government official, 27 October 2014	Kano, Nigeria	44	M
NGO representative, 28 October, 2014	Kano, Nigeria	45	F
NGO representative, 28 October, 2014	Kano, Nigeria	46	F
NGO representative, 29 October, 2014	Kano, Nigeria	47	F
State government official, 29 October 2014	Kano, Nigeria	48	M
NGO representative, 30 October, 2014	Kano, Nigeria	49	M
Educator, 30 October 2014	Kano, Nigeria	50	M
NGO representatives, 30 October 2014	Kano, Nigeria	51	F
NGO representative, 30 October, 2014	Kano, Nigeria	52	F

*Note*: A total of 52 interviews were conducted across Nigeria. All were numbered serially by date of interview

## References

[1] United Nations Population Fund (UNFPA). State of World Population 2013: Motherhood in Childhood--Facing the Challenge of Adolescent Pregnancy. New York: UNFPA; 2013.

[2] UNAIDS. Global AIDS Update Geneva: UNAIDS; 2016.

[3] United Nations. The Global Strategy for Women’s, Children’s and Adolescents’ Health (2016–2030). New York: Every Woman Every Child; 2015.

[4] Kirby D (2008). The impact of abstinence and comprehensive sex and STD/HIV education programs on adolescent sexual behavior. Sex Res Social Policy.

[5] Haberland N (2015). The case for addressing gender and power in sexuality and HIV education: a comprehensive review of evaluation studies. Int Perspect Sex Reprod Health.

[6] Cabezón C, Vigil P, Rojas I, Leiva M, Riquelme R, Aranda W, García C (2005). Adolescent pregnancy prevention: an abstinence-centered randomized controlled intervention in a Chilean public high school. J Adolesc Health.

[7] Kågesten A, Parekh J, Tunçalp Ö, Turke S, Blum RW (2014). Comprehensive adolescent health programs that include sexual and reproductive health services: a systematic review. Am J Public Health..

[8] Pick S, Givaudan M, Reich MR (2008). NGO-government partnerships for scaling up: sexuality education in Mexico. Dev Pract.

[9] Nove A (2014). Maternal mortality in adolescents compared with women of other ages: evidence from 144 countries. Lancet Glob Health.

[10] United Nations Educational, Scientific and Cultural Organization (UNESCO). Comprehensive Sexuality Education: The Challenges and Opportunities of Scaling-Up. Paris: UNESCO; 2012.

[11] Meyer JW, Boli J, Thomas GM, Ramirez FO (1997). World society and the nation-state. Am J Sociol.

[12] Zwingel S (2012). How do norms travel? Theorizing international women’s rights in transnational perspective. Int Stud Q.

[13] Mearsheimer J (1994). The false promise of international institutions. Int Secur.

[14] Katzenstein PJ (1996). The culture of national security: norms and identity in world politics.

[15] Finnemore M (1996). National interests in international society.

[16] Wendt A (1999). Social theory of international politics.

[17] Finnemore M, Sikkink K (1998). International norm dynamics and political change. Int Organ.

[18] Sewell WH (1992). A theory of structure: duality, agency, and transformation. Am J Sociol.

[19] Kim HJ, Sharman JC (2014). Accounts and accountability: corruption, human rights, and individual accountability norms. Int Organ.

[20] Cortell AP, Davis JW (2005). When norms clash: international norms, domestic practices, and Japan's internalisation of the GATT/WTO. Rev Int Stud..

[21] Keck ME, Sikkink K (1998). Activists beyond borders: advocacy networks in international politics.

[22] Elgström O (2000). Norm negotiations: the construction of new norms regarding gender and development in EU foreign aid policy. J Eur Public Policy..

[23] Acharya A (2004). How ideas spread: whose norms matter? Norm localization and institutional change in Asian regionalism. Int Organ.

[24] Sil R, Katzenstein PJ (2010). Beyond paradigms: analytic eclecticism in the study of world politics.

[25] Wendt A (1987). The agent-structure problem in international relations theory. Int Organ.

[26] Yin R. Case study research. 3^rd^ ed. Thousand Oaks: Sage; 2003.

[27] Collier D (2011). Understanding process tracing. PS Polit Sci Polit.

[28] Brady HE, Collier D (2010). Rethinking social inquiry: diverse tools, shared standards.

[29] Demehin AO (1983). Sex education in Nigeria: problems and proposals. Public Health..

[30] Adepoju A (2005). Sexuality Education in Nigeria: Evolution, Challenges and Prospects.

[31] Ibadan Social and Evaluation Research Team (ISERT). Evaluating the Implementation of Sexuality and Life Skills Education among In-School and Out-of-School Adolescents in Nigeria. Lagos: Ford Foundation; 2014.

[32] Robinson RS (2017). Intimate interventions in Global Health: family planning and HIV prevention in sub-Saharan Africa.

[33] Action Health Incorporated (AHI). The AHI Story 1989-2001. Lagos: Action Health Incorporated; 2002.

[34] Action Health Incorporated (AHI) (2002). Building Alliances for Sexuality Education.

[35] Brocato V. Establishing guidelines for comprehensive sexuality education: lessons and inspiration from Nigeria. New York: Sexuality Information and Education Council of the United States (SIECUS); 2005.

[36] SIECUS (1991). Guidelines for Comprehensive Sexuality Education: Kindergarten-12th Grade.

[37] Hodgson D, Watkins SC (1997). Feminists and neo-Malthusians: past and present alliances. Popul Dev Rev.

[38] World Bank (2008). Education and HIV/AIDS: A Sourcebook of HIV/AIDS Prevention Programs.

[39] MacArthur Foundation. A newsletter from The John D. And Catherine T. MacArthur Foundation. Chicago: MacArthur Foundation; Winter 2007, Vol. I.

[40] Federal Ministry of Health (2013). National HIV & AIDS and Reproductive Health Survey.

[41] Federal Ministry of Health (1995). National Adolescent Health Policy.

[42] Action Health Incorporated (AHI) (2002). A Unique Partnership for Adolescents’ Well Being in Nigeria.

[43] Action Health Incorporated (AHI) (2001). Nigerian Civil Society Organisations’ Dialogue on Comprehensive Sexuality Education.

[44] National Council on Education Abuja, Nigeria: National Council on Education; 1999.

[45] NERDC (2003). National Family Life and HIV education curriculum for junior secondary schools in Nigeria.

[46] Dlamini N, Okoro F, Ekhosuehi UO, Esiet A, Lowik AJ, Metcalfe K (2012). Empowering teachers to change youth practices: evaluating teacher delivery and responses to the FLHE programme in Edo state. Nigeria. Afr J Reprod Health..

[47] National Council on Education Abuja, Nigeria: National Council on Education; 2001.

[48] Wood SY, Rogow D (2015). Can Sexuality Education Advance Gender Equality and Strengthen Education Overall? Learning from Nigeria’s Family Life and HIV Education Program.

[49] Federal Ministry of Health, Action Health Incorporated (AHI). Assessment Report of the National Response to Young People’s Sexual and Reproductive Health in Nigeria. Abuja: Federal Ministry of Health; 2009.

[50] Federal Ministry of Education, Nigeria, Action Health Incorporated (AHI). Guidelines for Implementing the National Family Life and HIV Education (FLHE) Curriculum. Lagos: Federal Ministry of Education; 2008.

[51] National Commission for Colleges of Education (NCCE). Family life and emerging health issues curriculum: training guide for colleges of education in Nigeria. Abuja: National Commission for Colleges of Education; 2009.

[52] Oladeji A. et al. Global Fund support for FLHE implementation in Nigeria: innovation for achieving much with little. Paper presented at the seventh IAS conference on HIV pathogenesis and treatment, Kuala Lumpur, Malaysia; 2013.

[53] Action Health Incorporated (AHI). Foundation for a Healthy Adulthood: Lessons from School-Based Family Life and HIV Education Curriculum Implementation in Lagos State. Lagos: AHI and Lagos State Ministry of Education; 2010.

[54] United Nations Educational, Scientific and Cultural Organization (UNESCO) (2011). School-Based Sexuality Education Programmes: A Cost and Cost-Effectiveness Analysis in Six Countries.

[55] Esiet A, Esiet U, Philliber S, Philliber W (2009). Changes in knowledge and attitudes among junior secondary students exposed to the family life and HIV education curriculum in Lagos state. Nigeria Afr J Reproduc Health.

[56] National Population Commission (NPC) [Nigeria], ICF International. Nigeria Demographic and Health Survey 2013. Abuja, Nigeria and Rockville, Maryland, USA: NPC and ICF International; 2014.

[57] Guardian. PHD condemns sexuality education in schools. The Guardian. July 21, 2005.

[58] Bergstrom K (2002). Michigan State University Working paper # 276. Legacies of Colonialism and Islam for Hausa Women: An Historical Analysis, 1804–1960.

[59] Vaughan O, Banu SZ (2014). Muslim Women’s rights in northern Nigeria.

[60] Esiet U (2012). The Education Sector Response to HIV/AIDS in Nigeria: Status of FLHE Curriculum. Presentation.

[61] Barrett D, Kurzman C, Shanahan S (2010). For export only: diffusion professionals and population policy. Soc Forces.

[62] Robinson RS (2015). Population policy in sub-Saharan Africa: a case of both normative and coercive ties to the world polity. Popul Res Policy Rev.

[63] Pierson P, Mahoney J, Rueschemeyer D (2003). Big, slow-moving, and…invisible: Macrosocial processes in the study of comparative politics. Comparative historical analysis in the social sciences.

[64] Robinson RS, Kunnuji M, Shawar YR, Shiffman J. Prioritising sexuality education in Mississippi and Nigeria: the importance of local actors, policy windows and creative strategy. Glob Public Health. 2018; 10.1080/17441692.2018.1449000. 10.1080/17441692.2018.1449000 29557293

[65] Kunnuji M, Robinson RS, Shawar YR, Shiffman J (2017). Variable implementation of sexuality education in three Nigerian states. Stud Fam Plan.

